# Twentieth Century Reanalysis version 3 as a source of information on long-term trends (1806–2022) in lake surface water temperature changes in Central Europe (Poland)

**DOI:** 10.1038/s41598-025-28581-7

**Published:** 2025-12-15

**Authors:** Mariusz Ptak, Rajmund Przybylak, Przemysław Wyszyński, Mariusz Sojka

**Affiliations:** 1 Department of Hydrology and Water Management, Adam Mickiewicz University, Krygowskiego 10, 61-680 Poznań, Poland; 2 Faculty of Earth Sciences and Spatial Management, Nicolaus Copernicus University, Lwowska 1, 87-100 Toruń, Poland; 3 Centre for Climate Change Research, Nicolaus Copernicus University, Lwowska 1, 87-100 Toruń, Poland; 4 Department of Land Improvement, Environmental Development and Spatial Management, Poznań University of Life Sciences, Poznań, Piątkowska 94E, 60-649 Poznań, Poland

**Keywords:** Water temperature, Climatic change, Reconstruction, Trend, Poland, Climate sciences, Environmental sciences, Hydrology, Water resources

## Abstract

Water temperature is one of the fundamental characteristics of the hydrosphere, determining the functioning of its various components. In the case of lakes, surface water temperature shows a strong correlation with air temperature, and this relationship forms the basis for reconstructing the thermal regime of lakes. The study uses the Twentieth Century Reanalysis (20CRv3) meteorological dataset to reconstruct the surface water temperature of seven lakes in Poland for the period 1806–2022. This approach significantly expands the current state of knowledge, particularly for Central Europe, and includes periods predating significant human impact on the environment. Over the course of more than 200 years, an increase in water temperature has been observed, averaging 0.081 °C per decade across all studied lakes. Considering the changes in water temperature in the studied lakes, several distinct phases can be observed, which generally reflect changes in climatic conditions. Based on the results of the Pettitt test, the characteristic points include the 1840s, the 1940s, and the late 1980s. Rapid warming has been recorded in recent decades, and current studies suggest this trend is likely to continue in the future. This situation calls for multidisciplinary consultation and subsequent action to develop strategies for mitigating the impact of global warming on lake ecosystems.

**Supplementary Information:**

The online version contains supplementary material available at 10.1038/s41598-025-28581-7.

## Introduction

Knowledge about the natural environment depends on the duration and accuracy of measurements related to its individual components. One of the fundamental characteristics of the atmosphere is air temperature, which defines its thermal state and determines the rate and scale of many processes and phenomena occurring within the Earth’s climate system. Therefore, with the progress of civilization, instrumental measurements of air temperature were undertaken relatively early compared to other environmental studies^1–3^. Linking air temperature—and other climatic elements characterizing the atmosphere (precipitation, wind)—with other components of the environment (geosphere, biosphere, hydrosphere) enables the assessment of changes in those components over centuries. Particularly strong relationships are observed between the atmosphere and the hydrosphere. These connections are used to reconstruct gaps in the record of hydrological processes^4,5^. Owing to its physicochemical properties, water responds clearly to changes in air temperature. This fact is widely used in studies of inland water thermal regimes, even in the absence of other climatic data. These studies often yield highly accurate results in explaining changes in surface water temperature across various temporal scales^6,7^. In the case of lakes, water temperature observations were already being conducted in the 19th century^8^. However, systematic measurements have only been regularly recorded since the early 20th century^9^. Therefore, lake temperature records do not have time series as long as those for air temperature, which for about thirty stations in Europe have been recorded continuously since the 18th century. Against this background, there is a clear lack of sufficient knowledge about the long-term thermal changes in lakes.

At this point, it is important to highlight the fundamental role of water temperature in shaping the processes occurring in lakes^10–13^. In the long term, this influences the potential for using lakes for economic purposes such as irrigation, fisheries, tourism, and recreation. Today, a key issue is the response of lakes to climate change—an area of central importance in limnology— having detailed data allows for the interpretation of the magnitude of ecosystem changes occurring in lakes. In many regions around the world, a rise in surface water temperature has been observed, although the rate and scale of this warming vary^14,15^. Despite evidence of significant lake warming in recent decades, our understanding of long-term temperature changes remains relatively limited^9^. To fully understand the scope of the ongoing transformation in lake thermal characteristics over the past few decades, it is essential to collect information across various time scales. Expanding knowledge of lake temperature changes before the 20th century requires a reconstruction-based approach^16,17^.

Meteorological reanalysis is becoming an increasingly common tool for data acquisition and is widely used in various studies related to hydrological issues^18–20^. In the case of water temperature, that was demonstrated for Lake Chaohu^21^, the use of reanalysis combined with hydrodynamic models can provide valuable insights into its dynamics.

The main aim of the article is to reconstruct the annual and seasonal surface water temperature (LSWT) of selected lakes in Poland using the Twentieth Century Reanalysis (20CR) dataset. Based on the implementation of these assumptions, additional objectives were adopted, namely to determine the direction and magnitude of water temperature changes over the period 1806–2022. Achieving these objectives will provide an important starting point for further research on the thermal dynamics of inland waters, covering periods prior to significant human impact on the environment.

## Materials and methods

### Study area

**Fig. 1:**
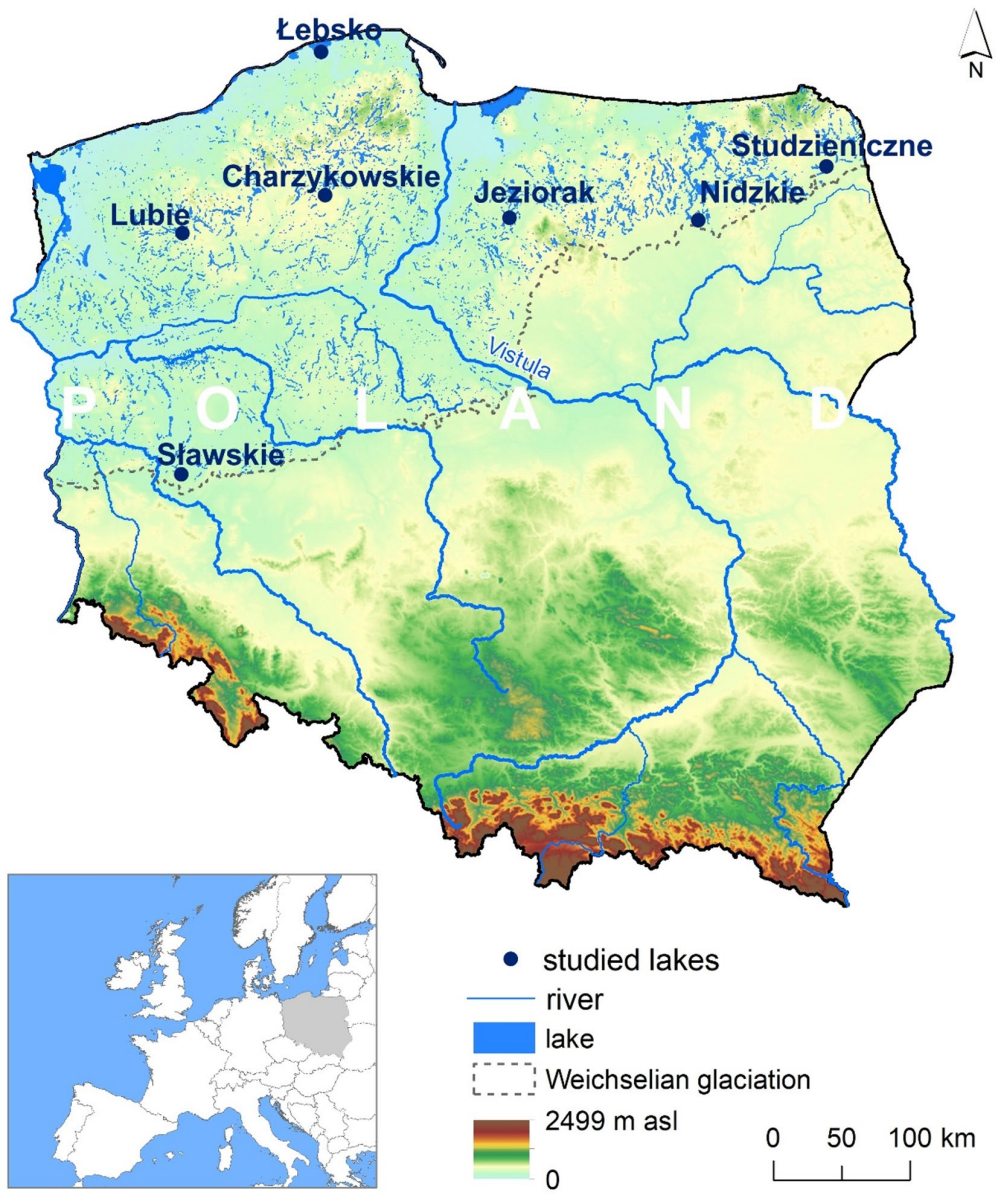
Studied lakes (figure generated in ArcGIS Pro v3.1.0 software), https://www.esri.com/en-us/arcgis/products/arcgis-pro/overview).

The study area covers lakes in the northern part of Poland (Fig. 1). The article analyses seven lakes, selected based on the availability of long-term surface water temperature measurements. All the lakes are of natural character and vary in morphometric parameters, with surface areas ranging from 2.44 to 70.20 km^2^ and mean depths from 1.6 to 11.6 m (Table 1). Notably, the geographical locations of the lakes place them under the influence of both maritime climate characteristics (western Poland) and continental climate features (eastern Poland). The mean air temperature ranges from 6.7 °C to 8.9 °C (east and west, respectively). In turn, the mean annual temperature of the analyzed lakes ranges from 9.3 °C (Łebsko) to 11 °C (Sławskie). The duration of the ice cover varies from 59 days (Lake Sławskie) to 96 days (Studzieniczne). Additionally, the northernmost Lake Łebsko is directly connected to the Baltic Sea, where one of the characteristics of coastal lakes is their shallow depth^22^. Cieśliński^23^, in determining the hydrochemical type of the water, points to dominant supply from chloride–sodium waters, indicating a constant influence of the Baltic Sea, where the average chloride concentration exceeds 750 mg dm⁻³.

**Table 1:** Location and basic morphometric parameters of the analyzed lakes^24^^*^, ^25^^**^.

No.	Lake	Latitude (deg *N*)	Longitude (deg E)	Altitude^*^ (m a.s.l.)	Area^*^ (km^2^)	Depth* (m)		Volume^*^ (10^6^ m^3^)	Transparency^**^ Secchi [m]
						Mean	Max		
1	Lubie	53.45	15.91	95.5	14.87	11.6	46.2	169.8	2.7
2	Sławskie	51.89	16.02	56.9	8.23	5.2	12.3	42.66	1.1
3	Łebsko	54.71	17.38	0.3	70.20	1.6	6.3	117.52	0.6
4	Charzykowskie	53.79	17.51	120	13.36	9.8	30.5	134.53	2.1
5	Jeziorak	53.72	19.61	99.2	31.53	4.1	12.9	141.59	0.8
6	Nidzkie	53.58	21.54	117.9	17.5	6.2	23.7	113.87	2.1
7	Studzieniczne	53.86	23.12	123.4	2.44	8.7	30.5	22.07	2.7

### Materials

Two datasets were used in this study. The first one pertains to surface water temperature, obtained from measurements conducted by the Institute of Meteorology and Water Management – National Research Institute over the past 63 years (1960–2022). The data was available for all the lakes. Water temperature is routinely measured at a fixed point, always at the same location, at a depth of 0.4 m below the surface, at 6:00 UTC.

The second one is the Twentieth-Century Reanalysis, version 3 (20CRv3^26^). 20CRv3 is a comprehensive historical global reanalysis dataset that provides a range of atmospheric variables, including, among others, 2-meter above-ground-level (a.g.l.) air temperature. It covers the time period from 1806 to 2015 with the spatial resolution of 1.0 degree latitude x 1.0 degree longitude global grid (360 × 181). To achieve this extended temporal coverage, the reanalysis assimilates solely surface pressure observations. A detailed overview of the 20CR system, including a technical description of the data assimilation and the model used, is provided by Slivinski et al.^26^. 20CRv3 is capable of reliably generating atmospheric estimates across a range of scales, from individual weather events to long-term climate trends^27^. The monthly mean air temperature data at a height of 2 m a.g.l. utilised in this study were extracted from the 20CRv3 grid point nearest to the location of the studied lake (refer to Table 1), employing the nearest-neighbour remapping technique.

Although 20CRv3 does not exhibit air temperature reconstruction biases for mid-latitudes when compared with other reanalyses for the contemporary period^27, their Fig. 10]^, we nevertheless conducted an evaluation of the 1 × 1° gridded temperature data against long-term historical point measurements from Polish meteorological stations (Fig. S1), i.e. Gdańsk 1851–1959^28^, Toruń 1871–1959^29^, Warszawa 1806–1959^30^. The available data range from the beginning of the measurements and/or the temporal coverage of 20CRv3 up to 1959, as this period of 20CRv3 gridded data was used as input for the reconstruction of lake water temperatures. The Pearson correlation (r) between the 20CRv3 gridded data and point measurements is very high (0.99) and statistically significant, with a coefficient of determination (R²) between 0.98 and 0.99, root mean square error (RMSE) of 0.73–1.16, and mean absolute error (MAE) of 0.55–0.86. Furthermore, when compared over the common period 1871–2015 for all stations (not shown), and with similarly high r and R² values, RMSE decreases to 0.63–0.73 and MAE to 0.48–0.54. Therefore, the use of 20CRv3 gridded data appears justified for reconstructing water temperature in lakes whose surface area is considerably smaller than that of a single grid cell.

However, it should be kept in mind that the air temperature in 20CRv3 is underestimated for the period 1806–1850 (the mean annual difference between observational data from Warszawa and 20CRv3 is − 1.0 °C, see Fig. S2). This is due to the limited assimilation of input data into 20CRv3 prior to the year 1850^26, their Fig. 1]^.

### Methods

In order to reconstruct the lake LSWT (Lake Surface Water Temperature) for the period 1806–1959, the air2water model was used. The air2water is a hybrid model that combines a physically-based equation (the surface layer energy balance) with stochastic calibration of the model parameters. Heat budget of the surface layer is calculated as follows:

1 $$\:\rho\:{C}_{p}V\frac{dLSWT}{dt}={AH}_{net}$$

where: ρ - water density (1000 kg m^− 3^), C_p_ - specific heat capacity at a constant pressure (4186 J kg^− 1 o^C^−1^), V - surface volume (m^3^), *LSWT* - lake surface water temperature (^o^C), *t* - time in days, A - surface area (m^2^), H_net_ - heat flux into the surface layer (W m^− 2^).

The air2water model has been successfully used to study LSWT in various regions around the world^7,31,32^. In this study, the 6-parameter version of the air2water model was applied^33^.

2 $$\:\frac{dLSWT}{dt}=\frac{1}{\delta\:}\left[{a}_{1}+{a}_{2}{T}_{air}-{a}_{3}LSWT+{a}_{c}\text{c}\text{o}\text{s}\left(2\pi\:\left(\frac{t}{{t}_{y}}-{a}_{6}\right)\right)\right]$$

3 $$\:\delta\:=\left\{\begin{array}{cc}exp\left(-\frac{LSWT-{T}_{h}}{{a}_{4}}\right)&\:LSWT\ge\:{T}_{h}\\\:1&\:LSWT<{T}_{h}\end{array}\right.$$

where: *T*_*air*_ - air temperature (^o^C), *a*_*1*_, *a*_*2*_, *a*_*3*_, *a*_*4*_, *a*_*5*_ and a_*6*_ *-* model parameters determined during the process of model calibration and validation, *t*_*y*_ - duration of a year (365 days), *T*_*h*_ - reference value of the deep-water temperature (^o^C), *δ* - dimensionless term representing the ratio between the volume of the surface lake layer and a reference volume.

**Fig. 2:**
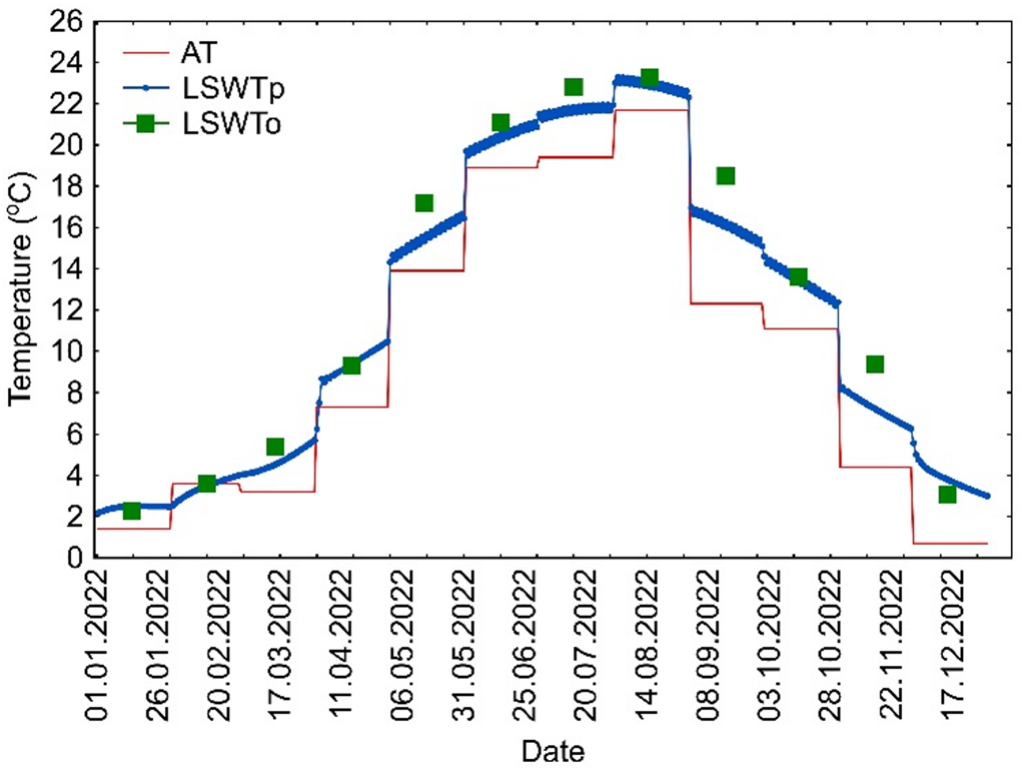
Approach to modeling lake water temperatures using the air2water program – example results from 2022 for Lake Sławskie (AT – air temperature, LSWTp – water temperature obtained from the air2water model, LSWTo – water temperature based on measurement data).

To assess the usefulness of the air2water model, it was calibrated using data from the period 1960–1999 (40 years – approx. 63%), while data from the period 2000–2022 (23 years – approx. 27%) were used for model validation. Since this study uses monthly average water and air temperatures, the input data for the air2water model were prepared so that each day was assigned the corresponding monthly average air and water temperature value (Fig. 2). Based on this data, the model was calibrated. For the validation, the average monthly LSWT values were compared with the LSWT values from the 15th day of each month.

To assess the performance of the model, six commonly used metrics were used, including coefficient of determination (R^2^), root mean square error (RMSE), mean absolute error (MAE), the Nash-Sutcliffe efficiency coefficient (NSE) and the Kling-Gupta efficiency coefficient (KGE).

To reconstruct the monthly average LSWT in the studied lakes for the period 1806–1959, the air2water model was calibrated individually for lakes. For this purpose, measurement data from the period 1960–2022 were used to determine the values of parameters *a1*,* a2*,* a3*,* a4*,* a5*, and *a6*. The values of R², RMSE, MAE, NSE, and KGE were recalculated and compared with the values obtained for the periods 1960–1999 (calibration) and 2000–2022 (validation using independent data).

Based on the reconstructed values of monthly average water temperatures, annual and seasonal averages were calculated for spring (March - May), summer (Jun - August), autumn (September - November), and winter (December - February). The Pettitt test allows for the detection of single change points in time series. To enable its use for detecting multiple change points within the 1806–2022 period, 500 time series of lengths 30, 40, and 50 years were randomly selected without replacement, and the Pettitt test was performed for each series. This way, statistically significant breakpoints (at the 0.05 significance level) were identified, allowing for the detection of years in which changes were most likely to have occurred, individually for each lake. To standardize the analysis periods across all studied lakes, a regional change point was determined. A given year was defined as the regional change point if a change was detected in 4 out of the 7 lakes. If the change was identified in two consecutive years, the earlier year was selected as the regional change point. The analysis of long-term changes was conducted for the entire 1806–2022 period and for sub-periods. Additionally, it was assumed that the minimum length of any analysis period must be at least 20 years. For the analysis of long-term changes, the Mann-Kendall^34^ and Sen’s^35^ tests were used. The Mann-Kendall and Sen’s tests were performed using a modified version of the mk package developed by Patakamuri and O’Brien^36^. The detection of change points was carried out using the trend package developed by Pohlert^37^. Trend analysis and change point detection were conducted using the R software environment (Version R-4.5.2 (https://cran.r-project.org/).

## Results

In the first stage, the air2water model was calibrated and validated using monthly average air temperature (AT) and LSWT. During the calibration stage, the following results were obtained: R² ranging from 0.981 to 0.992, RMSE from 0.63 to 0.95 °C, MAE from 0.50 to 0.76 °C, NSE from 0.878 to 0.922, and KGE from 0.959 to 0.994. Lower goodness-of-fit metrics were obtained during the model validation stage: R² ranged from 0.980 to 0.995; NSE from 0.853 to 0.910; and KGE from 0.913 to 0.979, while RMSE values were higher, ranging from 0.68 to 1.20 °C, and MAE from 0.54 to 0.94 °C (Table 2). The model performance evaluation results suggest that the air2water model can be reliably used to reconstruct data for the period from 1806 to 1959.

**Table 2:** The results of air2water model calibration (1960–1999) and validation (2000–2022).

Lake	Calibration (1960–1999)					Validation (2000–2022)				
	*R* ^2^	RMSE	MAE	NSE	KGE	*R* ^2^	RMSE	MAE	NSE	KGE
Lubie	0.981	0.95	0.76	0.878	0.981	0.980	1.20	0.94	0.853	0.913
Sławskie	0.992	0.66	0.51	0.922	0.990	0.992	0.83	0.69	0.899	0.949
Łebsko	0.992	0.63	0.50	0.921	0.994	0.990	0.68	0.54	0.910	0.979
Charzykowskie	0.990	0.69	0.54	0.914	0.986	0.987	0.89	0.65	0.898	0.943
Jeziorak	0.984	0.95	0.71	0.895	0.982	0.989	0.86	0.70	0.902	0.973
Nidzkie	0.991	0.72	0.56	0.917	0.982	0.995	0.79	0.66	0.908	0.948
Studzieniczne	0.989	0.82	0.62	0.907	0.959	0.992	0.77	0.64	0.908	0.947
**Minimum**	**0.981**	**0.63**	**0.50**	**0.878**	**0.959**	**0.980**	**0.68**	**0.54**	**0.853**	**0.913**
**Mean**	**0.988**	**0.77**	**0.60**	**0.908**	**0.982**	**0.989**	**0.86**	**0.69**	**0.897**	**0.950**
**Median**	**0.990**	**0.72**	**0.56**	**0.914**	**0.982**	**0.990**	**0.83**	**0.66**	**0.902**	**0.948**
**Maximum**	**0.992**	**0.95**	**0.76**	**0.922**	**0.994**	**0.995**	**1.20**	**0.94**	**0.910**	**0.979**

During the reconstruction of lake water temperatures for the years 1806–1959, the air2water model was calibrated using all available measurement data from the period 1960 to 2022. The calibration quality results of the air2water model are presented in Table 3. The following model fit statistics were obtained: R² ranging from 0.973 to 0.981, RMSE from 0.77 to 1.15 °C, MAE from 0.60 to 0.90 °C, NSE from 0.857 to 0.900, and KGE from 0.972 to 0.992.

**Table 3:** The results of air2water model calibration for the period of 1960–2022.

Lake	Calibration (1960–2022)				
	*R* ^2^	RMSE	MAE	NSE	KGE
Lubie	0.973	1.15	0.90	0.857	0.984
Sławskie	0.985	0.90	0.71	0.895	0.981
Łebsko	0.986	0.77	0.60	0.898	0.987
Charzykowskie	0.981	0.96	0.75	0.881	0.987
Jeziorak	0.982	1.02	0.78	0.888	0.990
Nidzkie	0.987	0.88	0.69	0.900	0.992
Studzieniczne	0.982	1.03	0.83	0.879	0.972
**Minimum**	**0.973**	**0.77**	**0.60**	**0.857**	**0.972**
**Mean**	**0.982**	**0.96**	**0.75**	**0.885**	**0.985**
**Median**	**0.982**	**0.96**	**0.75**	**0.888**	**0.987**
**Maximum**	**0.987**	**1.15**	**0.90**	**0.900**	**0.992**

**Fig. 3:**
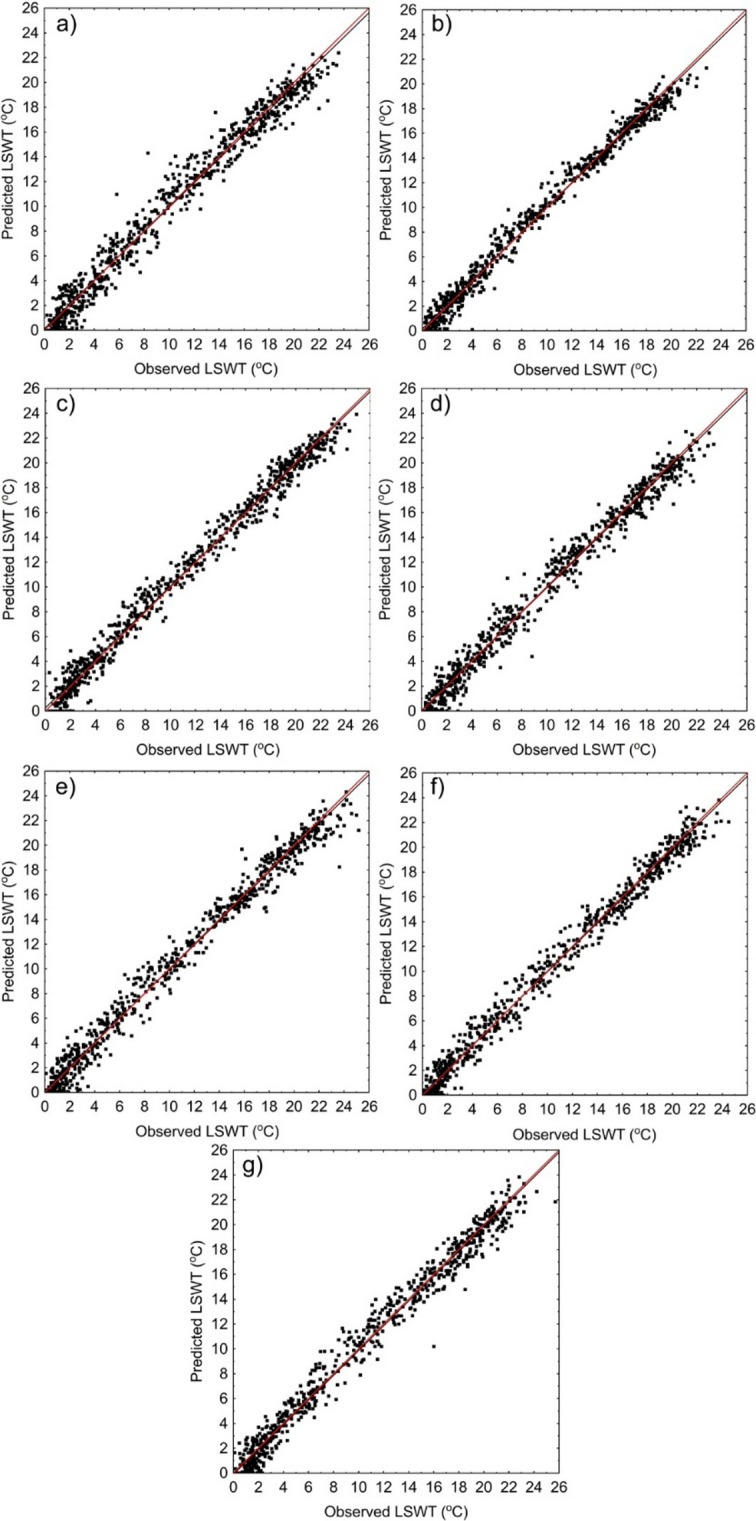
presents the observed LSWT in comparison to those obtained from the air2water model.

Figure 3 Predicted vs. observed LSWT for lakes Lubie (a), Łebsko (b, ) Sławskie (c), Charzykowskie (d), Jeziorak (e), Nidzkie (f) i Studzieniczne (g). The black line represents the fit between observed and predicted LSWT, whereas the red represents the case if all predicted values perfectly matched the observed ones.

The analysis of the annual average LSWT data series from 1806 to 2022 using Pettitt’s test revealed the presence of change points (Table S1). Between 9 and 12 change points were detected in the analyzed data series. In all lakes, changes were identified for the years 1987, 1998, and 2013; in six lakes for the years 1844 and 1988; and simultaneously in five lakes for the years 1909 and 1980. Pettitt’s test most frequently indicated a change point in the year 1844 (285 occurrences), followed by 1987 (191 occurrences). Additionally, in 1945, changes occurred 67 times across three lakes located in western Poland, and in 1988, changes were detected 88 times across six lakes.

**Fig. 4:**
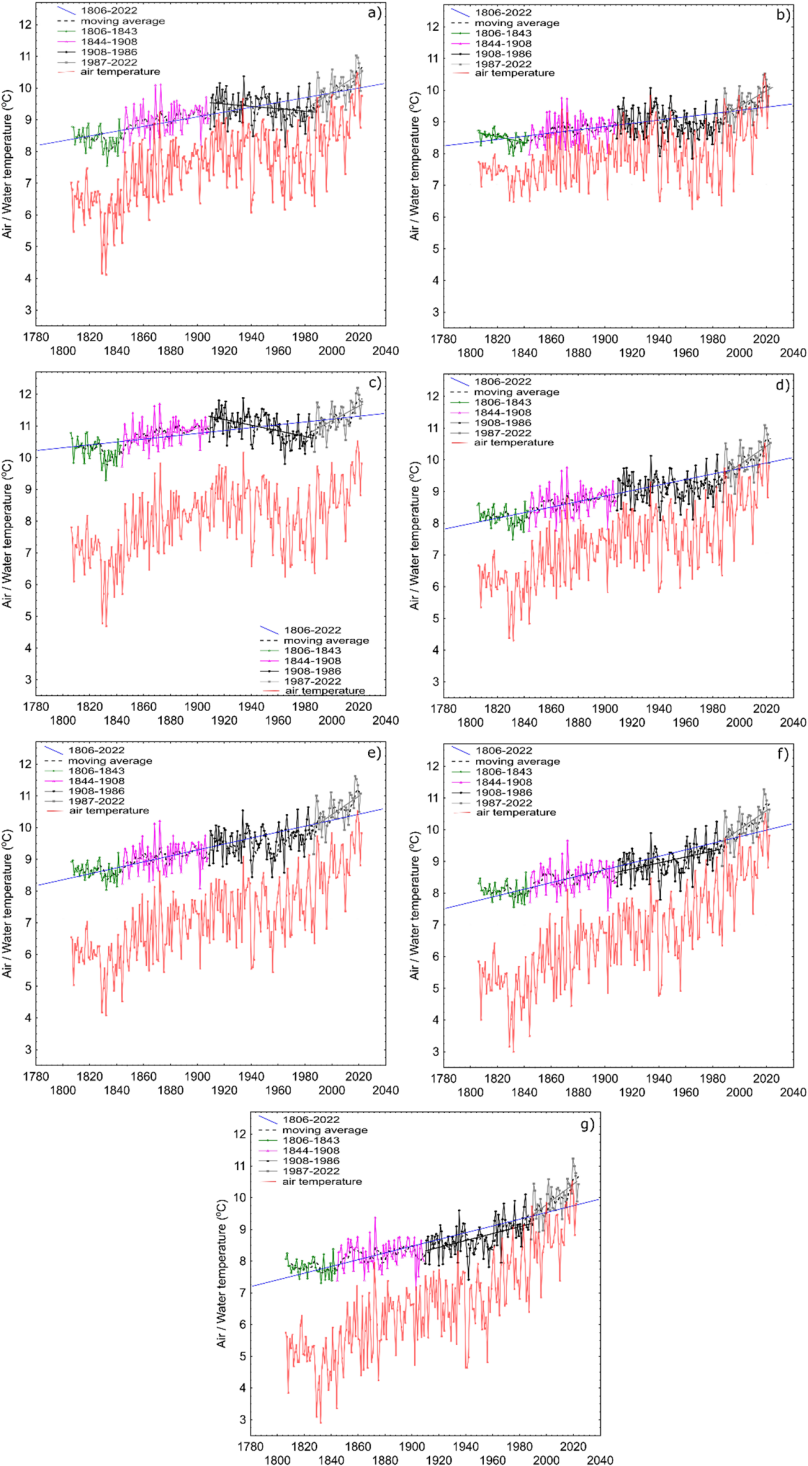
Results of the annual mean LSWT trend analysis for lakes Lubie (**a**), Łebsko (**b**), Sławskie (**c**), Charzykowskie (**d**), Jeziorak (**e**), Nidzkie (**f**), and Studzieniczne (**g**) (solid lines represent significant trends at a level of 0.05). Additionally, the year-to-year course in air temperature of the grid point closest to the lake was plotted based on data from of the 20CRv3^26^.

An analogous approach was applied for the spring, summer, autumn, and winter periods. The Pettitt test results revealed the presence of change points in different years (Tables S2–S5). Based on these results, so-called global change points were ultimately adopted, which allowed for the division of the data series into sub-series corresponding to the seasonal periods (Table S6). Based on the above assumptions, the analysis of changes in the average annual lake water temperatures was carried out using the Mann-Kendall and Sen’s tests for the periods 1806–1843 (38 years), 1844–1908 (65 years), 1909–1986 (78 years), and 1987–2022 (36 years) (Table 4). The average annual water temperatures in the lakes over the period 1806–2022 showed an increasing trend in all cases. The rate of temperature changes averaged 0.081 °C per decade, with the range of changes across individual cases varying from 0.049 to 1.05 °C per decade. Comparing values between two consecutive subperiods (determined based on Pettitt’s test analysis), an average temperature increase of 0.46 °C per decade was observed for 1806–1843 vs. 1844–1908; 0.40 °C per decade for 1844–1908 vs. 1909–1986; and as much as 0.81 °C per decade for 1909–1986 vs. 1987–2022 (Fig. 4).

**Table 4:** Trend analysis results. Bold values mean statistically significant trends.

Lake	S	Tau	z-value	Sen slope (°C/decade)	*p*-value
1806–2022					
Lubie	12,080	0.520	11.38	0.078	**0.000**
Sławskie	9374	0.404	8.83	0.049	**0.000**
Łebsko	9434	0.406	8.88	0.051	**0.000**
Charzykowskie	13,304	0.573	12.53	0.088	**0.000**
Jeziorak	13,710	0.590	12.91	0.096	**0.000**
Nidzkie	14,778	0.636	13.92	0.101	**0.000**
Studzieniczne	14,772	0.636	13.91	0.105	**0.000**
1806–1843					
Lubie	-78	-0.117	-1.01	-0.048	0.314
Sławskie	-138	-0.207	-1.79	-0.098	0.073
Łebsko	-220	-0.330	-2.86	-0.083	**0.004**
Charzykowskie	-76	-0.114	-0.98	-0.051	0.327
Jeziorak	-88	-0.132	-1.14	-0.045	0.255
Nidzkie	-84	-0.126	-1.09	-0.033	0.278
Studzieniczne	-90	-0.135	-1.16	-0.043	0.244
1844–1908					
Lubie	328	0.163	1.89	0.056	0.058
Sławskie	378	0.188	2.18	0.065	**0.029**
Łebsko	272	0.135	1.57	0.048	0.116
Charzykowskie	254	0.126	1.47	0.047	0.143
Jeziorak	260	0.129	1.50	0.041	0.133
Nidzkie	150	0.074	0.86	0.025	0.388
Studzieniczne	88	0.044	0.50	0.017	0.614
1909–1986					
Lubie	-466	-0.159	-2.05	-0.053	**0.041**
Sławskie	-966	-0.330	-4.24	-0.103	**0.000**
Łebsko	-376	-0.129	-1.65	-0.044	0.099
Charzykowskie	36	0.012	0.15	0.003	0.878
Jeziorak	296	0.101	1.30	0.031	0.194
Nidzkie	802	0.274	3.52	0.081	**0.000**
Studzieniczne	892	0.305	3.92	0.100	**0.000**
1987–2022					
Lubie	193	0.324	2.73	0.227	**0.006**
Sławskie	183	0.308	2.58	0.202	**0.010**
Łebsko	185	0.311	2.61	0.208	**0.009**
Charzykowskie	199	0.334	2.81	0.243	**0.005**
Jeziorak	199	0.334	2.81	0.236	**0.005**
Nidzkie	217	0.365	3.07	0.246	**0.002**
Studzieniczne	239	0.402	3.38	0.274	**0.001**

The analysis of average spring water temperatures in lakes for the period 1806–2022 using Pettitt’s test revealed breakpoints in the data series for 17 years. The most frequent breakpoints occurred in 1845 (287 times), 1980 (141 times), and 1988 (132 times). The analysis of spring water temperature averages using the Mann-Kendall test showed an overall significant increase from 1806 to 2022 (mean value of 0.15 °C per decade). The analysis of average lake water temperatures during the summer period revealed breakpoints in 19 different years. The most frequent breakpoints occurred in 1991 — 200 times, in 1841 and 1987 — 98 times each, and in 1931 — 90 times. The analysis of average summer water temperatures using the Mann-Kendall test showed a significant increasing trend over the period 1806–2022 (average 0.08 °C per decade). The analysis of the average lake water temperatures during the autumn period from 1806 to 2022 using the Pettitt test showed that breakpoints were identified in the data series for 14 years. The most frequent breakpoints occurred in 1998–175 times, and in 1959–82 times. Average autumn water temperatures showed an increase in six lakes (0.07 °C per decade), while in Lake Łebsko a decrease in water temperature was observed (0.02 °C per decade). Changes in other periods were statistically insignificant. Analysis of average water temperatures in lakes during the winter period from 1806 to 2022 using the Pettitt test revealed breakpoints in the data series for as many as 21 years. The most frequent breakpoints occurred in 1897–166 times, in 1969–123 times, and in 1987–78 times. Average winter water temperatures increased in six lakes (significant at the 0.01 significance level) over the period 1806–2022 (0.05 °C per decade), while in Lake Łebsko a decrease in water temperature was observed (0.02 °C per decade).

## Discussion

Understanding the processes occurring in lakes relies on a diverse set of methodologies^38–41^, one of which is the reconstruction of historical conditions. Research on the reconstruction of water temperature is gaining increasing interest^42–44^, driven by the need to create a broader understanding of thermal changes in the hydrosphere^45^. Depending on the adopted methodology and reference data, such analyses cover different time intervals. In relation to lakes, it should be emphasized that a large portion of thermal studies focuses on the last several decades^46–48^, while less attention has been given to periods reaching back to the first half of the 20th century^49,50^. The use of reanalysis data combined with hydrodynamic models can provide information on the dynamics of water temperature in individual water bodies^21^. In line with these findings, the reconstruction of water temperature in seven lakes in Poland presented in this article significantly expands the current knowledge on inland water thermal dynamics, covering a period of over 200 years.

Considering the changes in water temperature in the studied lakes, several distinct phases can be observed, which generally reflect shifts in climatic conditions. Based on the Pettitt test results, characteristic moments include the 1840s, the 1940s, and the late 1980s. The beginning of the analysed period is marked by the lowest temperatures and a decreasing trend. The first decades of the 19th century (up to 1840) in Poland were characterized by a greater degree of thermal continentality than that observed today. In a broader perspective, this represented the final phase of a climatic situation that had persisted since the 16th century^51^. From this point onward, water temperatures gradually increased, although some downward tendencies can be noted. One such period occurred from the mid-1940s, following one of the greatest warming phases of the 20th century, during which global temperatures rose by 0.37 °C between 1925 and 1944^52^. According to the Sen’s test, the key moment of thermal regime transformation occurred the late 1980s, when a marked warming of lake waters occurred relative to the preceding period. This change corresponds to a shift in the climatic regime, which also influenced lake temperatures. Similar observations were confirmed by previous studies conducted on 20 lakes in Central Europe^53^, where six lakes from the area of Poland were included. Furthermore, in two additional lakes (Studzieniczne and Białe Augustowskie, northeastern Poland), it was determined that a significant change occurred at the same time^54^.The results obtained in this study are consistent with other research on water temperature reconstructions. For example, over the past 150 years of inland water monitoring (Pannonian ecoregion, Europe), a clear warming trend has been observed, with most of the warming occurring in recent decades^55^. Significant changes over the last few decades are also evident in many other cases—for instance, the increase in Vrana Lake’s water temperature (Croatia) was particularly pronounced after 2013^56^. Trend analysis for Lake Miedwie (northwestern Poland) showed an average warming rate of 0.20 °C/decade, with the last thirty years of this period exhibiting an accelerated increase of 0.31 °C/decade^57^.

Throughout the entire analysis period from 1806 to 2022, the rate of change in water temperature varied widely, ranging from 0.049 °C per decade (Sławskie Lake) to 0.105 °C per decade (Studzieniczne Lake). Considering the extreme locations of these two cases—southwest and northeast Poland respectively—this variation should be explained by the characteristics of the regions where they are located. The northeastern part of Poland is characterized by a continental climate, one of whose distinctive features is colder and longer winters compared to the west. In relation to the hydrosphere, this translates into the duration of ice cover phenomena. This situation is changing with increasing global warming, resulting in a later onset of ice formation and an earlier ice break-up date. According to data collected for the period 1961–2010, the average duration of ice cover on Lake Sławskie was 59.2 days, whereas on Lake Studzieniczne it was over a month longer (96.4 days)^58^. Until recently, Lake Studzieniczne was effectively isolated from external (atmospheric) influences for one quarter of the year. The recorded changes in ice cover duration indicate that the rate of ice cover decreased by an average of 3.7 days per decade in the first case, and as much as 6.1 days per decade in the second^58^. Consequently, the increasingly shorter ice season leads to longer periods of water warming in lakes, which is reflected in a higher rate of increase in water temperature. The earlier onset of the stratification season in lakes was significant for heat storage and average surface water temperature^59^. Seasonal change analysis showed the highest increase during spring (0.15 °C/decade), which can also be attributed to the earlier ice cover break-up dates. Even in the 1960s, in many Polish lakes the average ice disappearance date fell in the third decade of March, while today it is at the end of February^60^. Furthermore, against the backdrop of seasonal data, a different response was recorded in Lake Łebsko to water temperature changes was observed compared to the other lakes. This situation is caused by two factors: depth and location. Lake Łebsko is a polymictic lake, similar to Lake Sławskie, in which, however, such seasonal reactions were not observed. Cieśliński and Chlost^61^ indicate that factors potentially influencing water temperature include the intensity of water exchange and the magnitude of marine water intrusions.

Considering the fundamental importance of water temperature for inland waters, the results obtained in this study should be regarded as unfavorable. The overall direction of changes observed over more than 200 years is unequivocal, indicating a permanent warming trend. This transformation poses a threat to lakes, causing disturbances in their functional balance. Increasing warming of the surface water layer will affect the stability of the water column. Yang et al.^62^ indicate that the development of thermal stratification is an important factor regulating the composition and abundance of phytoplankton during the summer period. As noted by Oleksy and Richardson^63^, an increase in the intensity and duration of stratification in dimictic lakes can alter the mixing regime of monomictic lakes, resulting in oxygen deficits in the hypolimnion, as well as changes in biogeochemistry and productivity. This concerns, among others, water quality issues, which is confirmed by studies such as those on Lake Yangzong (China), where water quality parameters were shown to be significantly correlated with and dependent on temperature^64^. Polish regulations concerning the classification and assessment of surface water bodies refer to the legal acts (directives) of the European Union, according to which the general status of the analyzed lakes is bad. Furthermore, the catchment areas of these lakes are sensitive to eutrophication, which leads, among other effects, to accelerated algae growth. Ongoing climate changes will increase the risk of cyanobacterial blooms in northern lakes, where in subarctic Quebec (Canada) the cyanobacterial community biovolumes positively correlated with surface water temperatures^65^. Similar situations have been observed in other regions; for example, in China, warming of Lake Dianchi’s surface temperature has extended the risk period for algal blooms and showed a positive correlation with algal density^66^. It should also be noted that water quality itself can influence water temperature. Previous analyses^23^ including the lakes currently under study, referred to these relationships by considering water transparency. PCA analysis showed negative relationships, where a decrease in transparency leads to an increase in water temperature due to greater absorption of solar radiation in the surface water layer.

In the context of the relationship between water temperature and its quality, oxygen concentration is a key factor, because it decreases with rising temperature. This limits the water’s self-purification capacity and affects aerobic organisms^67^. Many factors influence fish distribution, with water temperature and dissolved oxygen being particularly restrictive^68^. Research on Lake Tanganyika showed that climate warming and intensified stratification reduced the lake’s potential fish production, leading to decreased fish catches^69^. Large and deep lakes will likely serve as thermal refuges for cold- and cool-water fish species even as average lake temperatures rise^70^. Among the seven lakes analyzed, three have average depths not exceeding 6 m, and it is in these shallower lakes that the fastest changes in ichthyofauna composition due to rising temperatures are expected. Changes in thermal thresholds will be crucial for hydrobiological shifts. Potential gains in species numbers from warmer waters may not fully compensate for losses of cold-water species with ongoing warming^71^. Previous studies of inland water ichthyofauna in northern Poland indicate that species with upper thermal tolerance limits below 28 °C live at the edge of their range^72^. The observed changes over the past two centuries allow us to conclude that the last few decades are particularly alarming, with a marked increase in water temperature. According to current climate scenarios, the trends observed in recent years are expected to continue^54^. The scale of ongoing and anticipated future changes necessitates actions to mitigate the effects of lake ecosystem transformation.

## Conclusion

In the case of the hydrosphere, water temperature is one of its key parameters, with the distribution and changes in temperature influencing the functioning and transformation of its individual components. This article presents an analysis of the thermal regime of seven lakes in Central Europe over an unprecedented period spanning 1806–2022. The use of the air2water model, which utilizes air temperature data from the 20CRv3 reanalysis, proved to be an effective approach, as confirmed by high statistical test results. Overall, the observed changes reflect the prevailing climatic conditions. Across all cases, an increase in the average annual water temperature of 0.081 °C per decade in the period of 1806–2022 was recorded, with individual lakes exhibiting rates ranging from 0.049 to 0.105 °C per decade. Notably, the most significant increases were observed over the last few decades, and current studies suggest this warming trend will continue in the future. The results obtained in this study are unfavorable with regard to lake functioning, as the more than two-century-long warming will drive their transformation, contributing to declines in water quality and alterations in hydrobiological conditions. This situation calls for multidisciplinary consultations and subsequent actions aimed at developing strategies to mitigate the impacts of global warming on lake ecosystems.

## Supplementary Information

Below is the link to the electronic supplementary material.

Supplementary Material 1

## Data Availability

Datasets for this research were derived from the following public domain resources:- Lake Surface Water Temperature: Institute of Meteorology and Water Management – National Research Institute (IMGW-PIB) [https://danepubliczne.imgw.pl/data/dane\_pomiarowo\_obserwacyjne/dane\_hydrologiczne/] for the period 1984-2022 and data transcribed from Hydrological Yearbooks of IMGW-PIB, 1960-1983 by the first Author which are available on reasonable request.- Air Temperature: 20th Century Reanalysis (V3), [https://psl.noaa.gov/data/gridded/data.20thC\_ReanV3.html] . These data were produced by National Oceanic and Atmospheric Administration (NOAA) and are not subject to copyright protection in the United States. NOAA waives any potential copyright and related rights in these data worldwide through the Creative Commons Zero 1.0 Universal Public Domain Dedication (CC0-1.0).
