# Supplementary material for: Twentieth Century Reanalysis version 3 as a source of information on long-term trends (1806–2022) in lake surface water temperature changes in Central Europe (Poland)
Source: Sci Rep. 2025 Dec 15;15:43833. doi: 10.1038/s41598-025-28581-7 (PMC12706076; doi:10.1038/s41598-025-28581-7)
Supplement: Supplementary file 1 — Supplementary Material 1 [file 41598_2025_28581_MOESM1_ESM.docx]

**Supplementary material**

**
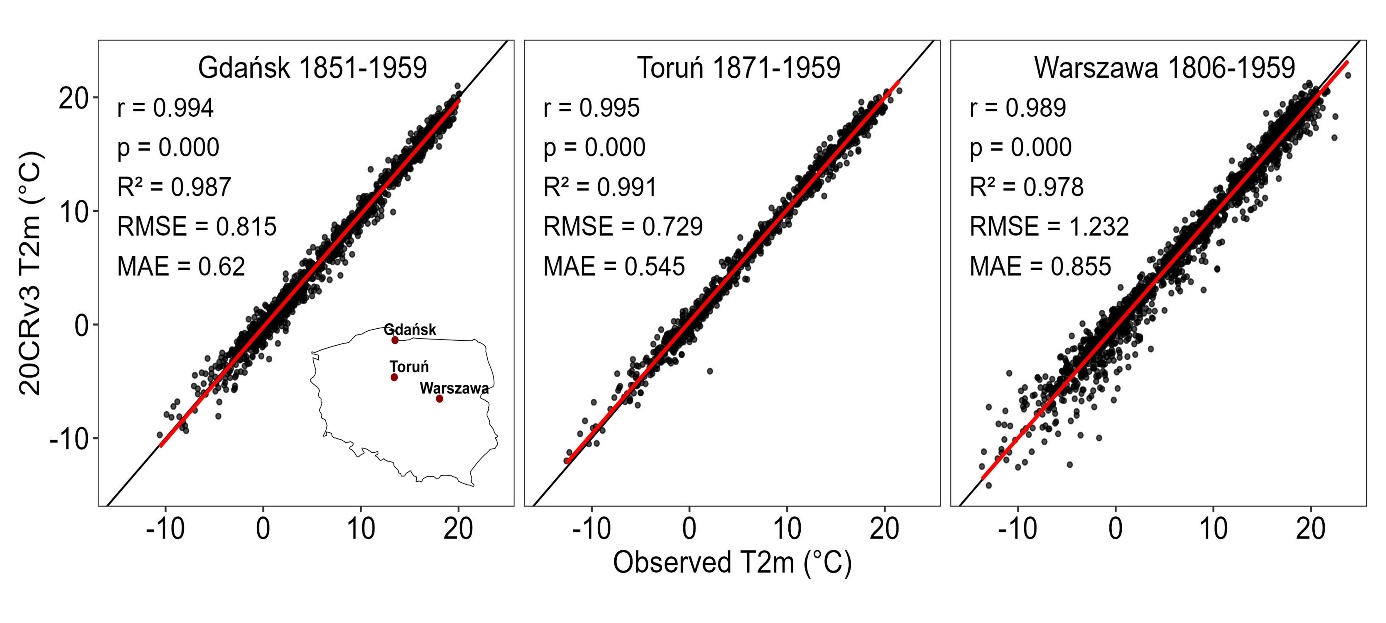
**

**Fig. S1.** Observed monthly average 2-meter above-ground-level air temperature (T2m, °C) from homogenised long term series for: Gdańsk (1851-1959), Toruń (1871-1959) and Warszawa (1806-1959) vs T2m taken from the nearest grid point from the 20CRv3 with the basic statistics: r – Pearson correlation coefficient, p – significance level, R^2^ – coefficient of determination, RMSE – root mean square error and MAE – mean absolute error. The red line illustrates how well the 20CRv3 aligns with the observed values, while the black line represents perfect agreement.

**
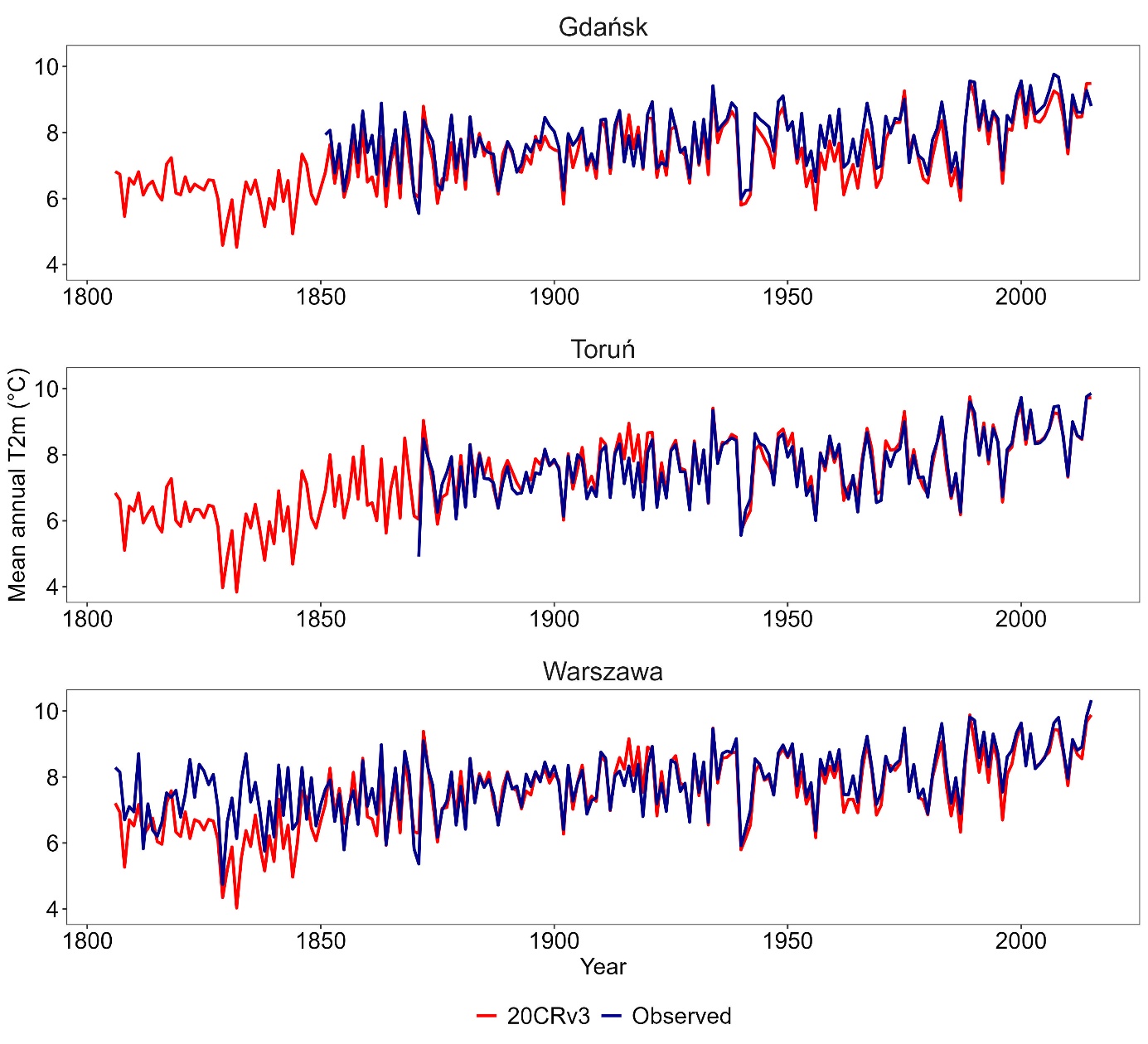
**

**Fig. S2.** Year-to-year courses of mean annual air temperature (T2m, °C) from observational data for Gdańsk, Toruń and Warszawa (blue line) as well as the air temperature from the nearest grid point taken from 20CRv3 (red line) in the period 1806-2015.

**Table S1**. Change points identified using Pettitt’s test (analysis of 500 random samples of 30, 40, and 50 years in length)

|  | Sławskie | Lubie | Łebsko | Charzykowskie | Jeziorak | Nidzkie | Studzieniczne | Year total |
| --- | --- | --- | --- | --- | --- | --- | --- | --- |
| 1827 | 11 | 2 | 14 |  |  |  |  | 27 |
| 1840 |  | 14 |  | 2 |  |  |  | 16 |
| 1844 | 6 | 43 |  | 55 | 57 | 62 | 62 | 285 |
| 1845 | 53 | 10 | 4 |  |  |  |  | 67 |
| 1850 | 4 |  | 6 |  |  |  |  | 10 |
| 1851 | 3 | 2 |  | 1 |  |  |  | 6 |
| 1856 |  |  | 5 |  |  |  |  | 5 |
| 1877 |  |  |  |  |  |  | 1 | 1 |
| 1909 | 23 | 15 |  | 8 | 1 | 10 |  | 57 |
| 1912 |  |  |  |  |  |  | 1 | 1 |
| 1939 |  |  |  |  |  |  | 59 | 59 |
| 1953 | 15 | 12 | 1 |  |  |  |  | 28 |
| 1958 |  |  |  |  |  | 6 |  | 6 |
| 1959 | 16 |  |  |  | 1 | 48 |  | 65 |
| 1961 | 31 |  |  |  |  |  |  | 31 |
| 1965 |  |  |  |  | 5 | 6 | 3 | 14 |
| 1980 |  |  | 1 | 10 | 14 | 8 | 7 | 40 |
| 1987 | 41 | 28 | 36 | 24 | 23 | 21 | 18 | 191 |
| 1988 |  | 15 | 6 | 16 | 15 | 18 | 18 | 88 |
| 1997 |  |  |  | 2 |  |  |  | 2 |
| 1998 | 7 | 8 | 8 | 6 | 9 | 9 | 14 | 61 |
| 2005 |  |  |  |  |  |  | 2 | 2 |
| 2013 | 3 | 2 | 2 | 2 | 2 | 2 | 1 | 14 |
| Lake total | 213 | 151 | 83 | 126 | 129 | 190 | 186 | 888 |

**Table S2.** Breakpoints in the series of average water temperatures from the spring period

| Spring | Sławskie | Lubie | Łebsko | Charzykowskie | Jeziorak | Nidzkie | Studzieniczne | Year total |
| --- | --- | --- | --- | --- | --- | --- | --- | --- |
| 1844 |  |  |  |  |  | 5 | 12 | 17 |
| 1845 | 43 | 49 | 7 | 57 | 46 | 48 | 37 | 287 |
| 1846 |  |  |  |  |  |  | 5 | 5 |
| 1849 |  |  |  |  |  |  | 1 | 1 |
| 1856 | 14 | 7 | 19 | 1 | 2 |  |  | 43 |
| 1858 |  |  | 3 |  | 3 |  |  | 6 |
| 1859 |  |  | 34 |  | 5 |  |  | 39 |
| 1881 | 1 |  |  | 2 |  | 5 | 10 | 18 |
| 1953 | 39 |  |  |  |  |  |  | 39 |
| 1958 |  |  |  |  |  | 19 | 26 | 45 |
| 1965 |  |  | 21 |  | 28 | 12 | 13 | 74 |
| 1966 |  |  | 2 | 8 |  | 1 | 8 | 19 |
| 1970 |  |  | 1 |  |  |  |  | 1 |
| 1980 | 12 | 14 | 18 | 26 | 28 | 23 | 20 | 141 |
| 1982 | 2 |  | 1 |  |  |  |  | 3 |
| 1987 | 26 | 3 | 16 | 6 |  |  |  | 51 |
| 1988 | 4 | 23 | 13 | 16 | 26 | 28 | 22 | 132 |
| Lake total | 141 | 96 | 135 | 116 | 138 | 141 | 154 |  |

**Table S3**. Breakpoints in the series of average water temperatures from the summer period

| Summer | Sławskie | Lubie | Łebsko | Charzykowskie | Jeziorak | Nidzkie | Studzieniczne | Year total |
| --- | --- | --- | --- | --- | --- | --- | --- | --- |
| 1841 | 22 | 31 |  | 15 | 14 | 12 | 4 | 98 |
| 1842 |  |  |  |  | 2 | 12 | 21 | 35 |
| 1844 |  | 1 |  |  |  | 11 | 13 | 25 |
| 1860 |  |  |  |  |  | 3 | 10 | 13 |
| 1861 |  |  |  |  |  |  | 1 | 1 |
| 1862 |  |  |  |  |  |  | 12 | 12 |
| 1928 | 10 |  |  |  |  |  |  | 10 |
| 1931 |  | 25 | 43 | 15 | 6 | 1 |  | 90 |
| 1939 |  |  | 1 |  |  |  |  | 1 |
| 1953 | 7 |  |  |  |  |  |  | 7 |
| 1958 |  |  | 44 |  | 9 | 3 |  | 56 |
| 1959 | 7 |  |  |  |  |  |  | 7 |
| 1962 |  |  | 27 |  | 22 | 16 |  | 65 |
| 1987 | 4 | 15 | 15 | 16 | 16 | 16 | 16 | 98 |
| 1991 | 29 | 29 | 27 | 29 | 28 | 28 | 30 | 200 |
| 1993 | 5 | 3 |  | 3 | 2 | 2 | 5 | 20 |
| 2000 | 2 | 2 | 4 | 2 | 4 | 4 | 1 | 19 |
| 2001 | 1 | 2 | 3 | 2 | 2 | 2 | 1 | 13 |
| 2012 | 1 |  | 1 |  |  |  |  | 2 |
| Lake total | 88 | 108 | 165 | 82 | 105 | 110 | 114 |  |

**Table S4**. Breakpoints in the series of average water temperatures from the autumn period

| Autumn | Sławskie | Lubie | Łebsko | Charzykowskie | Jeziorak | Nidzkie | Studzieniczne | Year total |
| --- | --- | --- | --- | --- | --- | --- | --- | --- |
| 1836 |  | 1 |  |  |  |  |  | 1 |
| 1838 |  | 2 |  |  |  |  |  | 2 |
| 1842 |  | 2 |  |  |  |  |  | 2 |
| 1850 |  |  |  |  |  | 5 | 3 | 8 |
| 1851 |  | 8 |  | 4 | 14 |  |  | 26 |
| 1922 |  |  |  |  |  |  | 1 | 1 |
| 1951 |  |  | 2 |  |  |  |  | 2 |
| 1959 |  |  | 42 |  |  | 9 | 31 | 82 |
| 1961 | 4 |  | 18 |  |  |  |  | 22 |
| 1963 | 31 | 1 | 21 |  |  |  |  | 53 |
| 1969 | 2 |  |  |  |  |  |  | 2 |
| 1998 | 19 | 27 | 26 | 27 | 25 | 25 | 26 | 175 |
| 2010 |  | 1 |  | 1 |  |  |  | 2 |
| 2011 | 1 |  |  |  |  |  |  | 1 |
| Lake total | 57 | 42 | 109 | 32 | 39 | 39 | 61 |  |

**Table S5**. Breakpoints in the series of average water temperatures from the winter period

| Winter | Sławskie | Lubie | Łebsko | Charzykowskie | Jeziorak | Nidzkie | Studzieniczne | Year total |
| --- | --- | --- | --- | --- | --- | --- | --- | --- |
| 1825 |  |  | 21 |  |  |  |  | 21 |
| 1828 | 8 |  |  |  |  |  |  | 8 |
| 1840 |  | 5 |  |  |  |  |  | 5 |
| 1841 | 4 |  |  |  |  |  |  | 4 |
| 1849 | 19 | 3 |  | 3 |  |  |  | 25 |
| 1850 |  |  | 35 |  |  |  |  | 35 |
| 1855 |  |  | 4 |  |  |  |  | 4 |
| 1897 | 36 | 44 | 32 | 37 | 17 |  |  | 166 |
| 1909 | 5 | 5 |  | 4 | 9 | 3 | 2 | 28 |
| 1939 |  |  | 1 |  |  |  |  | 1 |
| 1952 |  |  | 10 |  | 3 |  |  | 13 |
| 1959 | 11 |  | 49 |  |  |  |  | 60 |
| 1960 | 17 | 21 | 19 | 3 |  |  |  | 60 |
| 1961 | 5 |  | 2 | 5 |  |  |  | 12 |
| 1969 |  |  |  |  | 19 | 47 | 57 | 123 |
| 1970 | 1 | 1 | 2 | 1 |  |  |  | 5 |
| 1981 |  |  |  |  | 7 | 7 | 4 | 18 |
| 1982 | 2 | 30 | 7 | 35 |  |  |  | 74 |
| 1985 |  |  |  |  | 12 |  |  | 12 |
| 1987 | 27 | 10 | 22 | 10 | 9 |  |  | 78 |
| 2004 |  |  |  |  |  |  | 1 | 1 |
| Lake total | 135 | 119 | 204 | 98 | 76 | 57 | 64 |  |

**Table S6**. Division of the data series into sub-series based on the results obtained from Pettitt’s test

| Season | Subperiod 1 | Subperiod 2 | Subperiod 3 | Subperiod 4 | Subperiod 5 |
| --- | --- | --- | --- | --- | --- |
| Spring | 1806-1844 | 1845-1880 | 1881-1964 | 1965-1986 | 1987-2022 |
| Summer | 1806-1840 | 1841-1930 | 1931-1986 |  | 1987-2022 |
| Autumn | 1806-1849 | 1850-1997 |  |  | 1998-2022 |
| Winter | 1806-1848 | 1849-1896 | 1897-1959 | 1960-1986 | 1987-2022 |
| Year | 1806-1843 | 1844-1908 | 1909-1986 |  | 1987-2022 |
